# Sharing and Reuse of Sensitive Data and Samples: Supporting Researchers in Identifying Ethical and Legal Requirements

**DOI:** 10.1089/bio.2015.0014

**Published:** 2015-08-01

**Authors:** Murat Sariyar, Irene Schluender, Carol Smee, Stephanie Suhr

**Affiliations:** ^1^ Institute of Pathology, Charite—University Medicine Berlin, Berlin, Germany.; ^2^ TMF—Technologie und Methodenplattform e. V., Berlin, Germany.; ^3^ The Wellcome Trust Sanger Institute, UK ELIXER Hub, Hinxton, Cambridge, United Kingdom.; ^4^ European Molecular Biology Laboratory–European Bioinformatics Institute, UK ELIXER Hub, Hinxton, Cambridge, United Kingdom.

## Abstract

Availability of and access to data and biosamples are essential in medical and translational research, where their reuse and repurposing by the wider research community can maximize their value and accelerate discovery. However, sharing human-related data or samples is complicated by ethical, legal, and social sensitivities. The specific ethical and legal requirements linked to sensitive data are often unfamiliar to life science researchers who, faced with vast amounts of complex, fragmented, and sometimes even contradictory information, may not feel competent to navigate through it. In this case, the impulse may be not to share the data in order to safeguard against unintentional misuse. Consequently, helping data providers to identify relevant ethical and legal requirements and how they might address them is an essential and frequently neglected step in removing possible hurdles to data and sample sharing in the life sciences. Here, we describe the complex regulatory context and discuss relevant online tools—one which the authors co-developed—targeted at assisting providers of sensitive data or biosamples with ethical and legal questions. The main results are (1) that the different approaches of the tools assume different user needs and prior knowledge of ethical and legal requirements, affecting how a service is designed and its usefulness, (2) that there is much potential for collaboration between tool providers, and (3) that enriched annotations of services (e.g., update status, completeness of information, and disclaimers) would increase their value and facilitate quick assessment by users. Further, there is still work to do with respect to providing researchers using sensitive data or samples with truly ‘useful’ tools that do not require pre-existing, in-depth knowledge of legal and ethical requirements or time to delve into the details. Ultimately, separate resources, maintained by experts familiar with the respective fields of research, may be needed while—in the longer term—harmonization and increase in ease of use will be very desirable.

## Introduction

Efforts to make data and/or samples available and accessible so that they may be reused and repurposed in different contexts by the wider research community are gaining momentum. See, for example, the following funder policies: European Commission (EC) policy on open science data,^1^ National Institutes of Health (NIH) Sharing Policies and Related Guidance on NIH-Funded Research Resources,^2^ Wellcome Trust Data Sharing Policy,^3^ and Medical Research Council (MRC) Data Sharing Policy.^4^ The importance of data integration and sharing of biosamples across national borders^5^ is echoed by recent legal and ethical recommendations,^6,7^ for example, the ‘Framework for Responsible Sharing of Genomic and Health-Related Data’ published by the Global Alliance for Genomics and Health.^8^

To address the health and societal challenges of today, life science research, especially in clinical settings, increasingly requires the analysis of sensitive personal data and samples. At the same time, to increase the utility of these limited resources, there is a growing will to make data easily available and accessible, for example, to better enable the characterization of diseases and pathogens by increasing profiling capacities, for example, based on ‘omics’-data.^9–11^ Such characterizations lead to an increased granularity of disease profiles, which in turn reduces the number of patients per defined disease. In order to have sufficient numbers of patients for biomedical trials and research studies, and therefore increase the power of data analyses,^12,13^ collaboration and data sharing are necessary.

Although there are benefits to sharing patient-related data, for example, in the case of personalized medicine,^14,15^ there is a growing understanding of the risks related to data sharing:^16^ for example, disclosure of identifying biomedical data that can stigmatize or discriminate individuals and/or populations,^17^ or disclosure of hitherto unknown health risks to individuals who are neither prepared to receive this information, nor have access to appropriate medical counselling. In general, as soon as human data are involved, privacy and confidentiality become major issues. National laws and regulations as well as international legal requirements, such as the European Directive on Data Protection,^18^ mandate strict protection of personal identifiable data, which may include research results. The protection requirements for such data require careful thought and discussion in order for studies to meet their data-sharing objectives. For example, international projects such as the International Cancer Genome Consortium (ICGC) are committed to “mak[ing] the data available to the entire research community.”^19^ The trade-off between maximizing data sharing while minimizing possible risks to research participants' privacy is a key consideration.

While significant thought, effort, and resources have been and are being devoted to addressing organizational and technical hurdles^20^ that may prevent sharing and integration of data—for example, to identify appropriate repositories and make sure data complies with prevalent standards and formats—less help is available for providers of data or samples who may need advice prior to sample/data collection regarding legal and ethical considerations. Whether they are individual researchers, small research institutions, or large European research infrastructures, providers are faced with a complex and fragmented regulatory landscape concerning legal and ethical aspects. To maximize sharing of these valuable resources, providers of data or samples must receive adequate support in negotiating the technical and regulatory hurdles. Online resources can never replace detailed advice from experts but they can, for example, raise researchers' awareness of the need to consult the responsible research ethics committee to get information about specific local requirements.

One example of sample sharing is provided by the German Cooperative Health Research in the Region of Augsburg (KORA) project. Here, the KORA-gen resource for genetic epidemiological research is available,^21^ which harbors biosamples and phenotypic characteristics as well as environmental parameters of 18,000 adults from Augsburg and surrounding counties (for rules of access see ww.gsf.de/KORA-gen). The typical user of the KORA-gen resource is assumed to be located in Germany. If KORA-gen receives a request for kidney cancer-related biosamples from a researcher who is located (for example) in the UK, it has to consider the more general European framework. In such a case, it can be helpful to have a tool that lists relevant requirements and potential problems before processing the request further.

An example related to data sharing is “The Cancer Genome Atlas” (TCGA) data portal, which provides a platform for researchers to search, download, and analyze data sets generated by TCGA. It contains clinical information, genomic characterization data, and high level sequence analysis of tumor genomes (see https://tcga-data.nci.nih.gov/tcga/tcgaHome2.jsp).

European researchers who wish to contribute to such repositories should take account of legal and ethical issues before taking further steps. The following statement by the DNA Data Bank of Japan with respect to data submissions makes clear that such considerations have ubiquitous relevance:^22^ “For all data from human subjects researchers submitted to DDBJ, it is the submitter's responsibility to ensure that the privacy of a participant (human subject) is protected in accordance with all applicable laws, regulations and policies of the submitter's institute.”

Different online tools and services intended to aid sample/data providers in navigating legal and ethical requirements related to the sharing of sensitive data and samples were discussed during a one-day workshop in Berlin in June 2014, which was organized as part of the EU FP7-funded BioMedBridges project. Tools and services were selected based on their actual and potential relevance in the European research context. Here, we provide an overview of the challenges in making sensitive data and samples available, describe tools (one which the authors co-developed), and refer to some of the points raised in the workshop.

## The European Regulatory Context for Data and Sample Sharing

The EU Data Protection Directive 95/46/EC provides a detailed legal framework to which European researchers must adhere.^23^ A “Directive” in general provides a framework for Member States to adopt, but they may transpose the requirements of the Directive into their own national legislation. According to the European Court of Justice, a Directive becomes directly applicable if a State does not implement the contents of the Directive within the given time frame, or if the national implementation does not fulfil its requirements to the necessary level of precision. The main provision for biomedical research is in Article 8 of the EU Data Protection Directive, which imposes an enhanced level of protection on “special categories” of data, including health data. In addition, the Directive contains a list of definitions for key terms including “personal data” and “consent.” Anonymous data are not protected, while “pseudonymized data” (linked-anonymized data) remain personal data at least for the data controller who has access to the key or cipher.

Despite the strong common legal basis for EU Member States provided by the Directive, there is a perception among the research community that national data protection regulations make data sharing beyond borders difficult, time consuming or, in some circumstances, even impossible. The fragmentation of national regulations has three primary causes: first, legal documents are very concise, leaving considerable leeway concerning the interpretation of key terms such as “specific purpose,” “informed consent” or “anonymized data.” Second, even though exemptions from the consent principle are limited to cases of “substantial public interest,” Member States are free to define the precise meaning of this notion in different ways. Finally, Member States are free to provide for levels of data protection that are more stringent than required by the Directive. All this has led to data protection legislation which differs between European countries, a situation which is not likely to be changed by the General Data Protection Regulation as proposed by the European Commission and amended by the European Parliament^24^ since the aforementioned crucial questions remain unresolved (for further details on this issue, see Ref. 25).

In light of differing national legislation and considering the requirements of the EU Data Protection Directive, data sharing across all EU member states may only be considered permissible if data is unlinked anonymized or the Data Subject has given specific consent for the use of their (personal) data for the intended use. Two topics are important in the further discussion of the relevant legislation: anonymization and informed consent.

### Anonymization

With respect to anonymization there are three important considerations. First, it is very challenging to provide a precise definition of the term. In fact, terminology varies among EU Member States. In the UK for example, “linked-anonymized” or “pseudonymized” data may be treated as “anonymous” if the data controller does not have access to the linkage key. At the EU-level, the document entitled “Opinion 05/2014 on Anonymisation Techniques” of the Article 29 Data Protection Working Party^26^ leaves the question open while, in Germany, the Federal Court of Justice submitted the issue to the European Court of Justice in October 2014.^27^

Second, in the case of patient-related data or biosamples, it is unclear to what extent these can indeed be anonymized and, if they are anonymized, whether some of their value for research is lost.^28–31^ The former issue is related to risks of re-identification, especially when anonymized data is re-purposed in many different contexts. The value of data for research may be inversely connected to this: while using data of limited detail may lower the risk of re-identification, it may also affect the scientific value of the data. Although many would argue that the DNA sequence alone does not disclose the identity of an individual, it is increasingly acknowledged that the sharing of detailed genetic data unique to one person, such as whole genome sequence data, or the combination of datasets to enrich data, increases the risk of re-identification of the sample donor. Lin et al.^32^ reported that single base changes in 30 to 80 locations in the genome are sufficient to identify a single individual from a population of 10 billion. Arguably, the number of locations required for such re-identification might even be reduced further. For further legal and ethical impacts of whole genome sequencing, see Ref. 33 and the Public Health Genomics (PHG) Foundation's ‘Next Steps in the Sequence’.^34^

Third, full (unlinked) anonymization both deprives the donor of the possibility to use their right to withdraw consent, as well as making the return of research results or incidental findings impossible.

### Informed consent

Informed consent is an established approach to enable law-compliant processing of personal data and samples. With regard to obtaining consent or assessing whether pre-existing consent is sufficient, it has to be noted that truly *informed* consent should, by its very nature, explicitly indicate a specific research purpose, where the use of data or samples is limited to one single study with a clearly outlined study protocol. This specification can, however, be a challenge for some areas of biomedical research and even more so for biobanks. It also decreases the utility of data or samples and can remove them from the wider research context as re-use for purposes other than the original study to which the donor consented would be excluded.

To address this, the concept of *broad consent* is widely used for biobanks since the aim of a biobank can rarely be limited to a specific research project. While some EU member states do not consider broad consent as a valid form of consent, other countries use it without major concerns. In addition, national legislation may allow the use of samples in projects for which specific consent was not obtained from the sample donor. For example, the Human Tissue Act of England, Wales, and Northern Ireland,^35^ allows the use in research of archived, anonymized (linked or fully anonymized) human samples taken from living persons (living at the time the sample was taken) without specific consent, as long as the research study in question has been approved by a National Research Ethics Service (NRES) Research Ethics Committee (REC). It is important to note that sometimes the concept of a “biobank” varies between countries, which can cause some confusion when data and samples are to be shared across international boundaries. Biobanks in Finland, for example, may be discipline-specific, such as for hematology. While the new Finnish Biobanking Act allows reuse of samples and “data generated from them” with appropriate donor consent, it limits this reuse to studies within the specialty area of the biobank. It will be interesting to observe how this will play out in practice.

### Supporting researchers in navigating the legal landscape

For any tool or service that intends to provide researchers with solutions regarding data protection in international research projects, the fragmentation of regulation leads to questions about how legal issues should be addressed. There are two plausible options: (a) one could try to achieve a solution that will work in as many relevant jurisdictions as possible. Even with a huge amount of resources, full and detailed coverage of all possible use cases would hardly be possible without having a personal expert's advice service. (b) Efforts can be focused on the most likely use cases and the tool could point to the most conservative solution, for example, based on European law (i.e., the EU Directive on Data Protection) while highlighting possible exemption clauses in the applicable national jurisdiction for the case in question. The tools presented here choose different weightings between these two options.

### Online tools

We selected four tools that provide support with certain aspects of the complex regulatory landscape described above based on their relevance for the European research context. While three of the tools focus entirely on the European legal environment, the fourth—the International Policy interoperability and data Access Clearinghouse, IPAC—was included as an example of a resource that takes a wider, international approach. During a one-day workshop held in Berlin in June 2014, developers, legal experts, scientists, and IT experts debated how these tools could best help researchers in navigating local, national, European, and international legislation. The tools discussed at the workshop were:

• BioMedBridges Legal Assessment Tool (LAT)^36^
• Biobanking and Biomolecular Resources Research Infrastructure (BBMRI) legal WIKI^37^
• Human Sample Exchange Regulation Navigator (hSERN)^38^
• ‘International Policy interoperability and data Access Clearinghouse’ (IPAC) provided by the Public Population Project in Genomics and Society (P3G)^39^

To ensure that no other available resources were overlooked, a PubMed search was conducted using subsets of the keyword set {“data sharing”, “legal requirements”, “data protection”, “resources”, “tool”, “data privacy”}. Retired projects such as caBIG (cancer Biomedical Informatics Grid) or PrimeLife developed frameworks and system architectures that aimed at facilitating data sharing in light of data protection and privacy issues; however, as these resources are no longer actively maintained, they were not considered. Of course, there are other text-based resources available online, an example being the guide for “Publishing and sharing sensitive data” by the Australian National Data Service.^40^

### BioMedBridges Legal Assessment Tool (LAT)

This tool was co-developed by authors of this article(MS, IS) within the EU FP7-funded BioMedBridges project (http://www.biomedbridges.eu/workpackages/wp5). The primary purpose of LAT is to support data and sample providers and present the regulatory requirements for sharing sensitive data and/or samples. This is achieved by guiding the user through a structured query within a web-based graphical user interface: users are presented with a set of specific, relevant questions about the data and/or biosamples they wish to share and their specific use case. The user indicates certain characteristics that in turn drive underlying ethical and legal requirements: for example, the category (metadata, text data, images, genetic data, biosamples, or biosample associated data), the extent of disclosure (pseudonymous or anonymous), or the level of use restrictions (for example, intellectual property requirements). At the end of the query process, requirements, related information, solutions for fulfilling them and related templates are provided (see also http://www.biomedbridges.eu/sharing-sensitive-data). Due to the scope of BioMedBridges, the LAT is, in the first instance, not intended for use with studies involving new patients or donors, but rather for ongoing research projects with samples or data that may be re-used within different contexts and shared on a European level.

Even though the first version of the tool includes mainly EU-related requirements, the underlying requirement matrix can be extended to cover other specific national requirements (although this may require adjustments to the workflow, especially when new attributes are used to describe the requirements). Importantly, the BioMedBridges LAT does not assume any previous legal and/or ethical expertise on the side of the users, who may be early career researchers with no prior knowledge or awareness of legal and ethical requirements, experienced researchers who have not worked with sensitive data before, or researchers who regularly work with sensitive data but are planning to use it in a different research context or for a new purpose. Consequently, the tool assists many kinds of researchers who want to share sensitive data.

### BBMRI legal WIKI

The BBMRI legal WIKI-platform (http://www.bbmri-wp4.eu/wiki/index.php/Main_Page) is a knowledge platform intended to enhance the embedding of pan-European Biobanking in a European legal framework. The WIKI provides knowledge and documents/templates, and allows ‘grassroots’ contributions. It is currently kept up to date under the BBMRI-LPC (Biobanking and Biomolecular Resources Research Infrastructure–Large Prospective Cohorts) project umbrella. A login is required for the user to be able to browse beyond the main pages. The WIKI includes information about home state compliance with EU regulations; the information is grouped by nation, and then by topic. National biobank information is to some extent provided in different languages (for example, see http://www.bbmri-wp4.eu/wiki/index.php/Netherlands).

### Human Sample Exchange Regulation Navigator (hSERN)

The Human Sample Exchange Regulation Navigator provides users with structured information on the theoretical and practical legal requirements for exchanging biological samples across borders. The information is presented as follows (for more details, see http://www.hsern.eu/):

• Overview: provides a general comment related to the selected countries
• Theory: provides easy access to different legal notions and to the implemented legal texts (i.e., theoretical information on regulations)
• Practice: provides access to the legal or administrative forms and advises on the actions to undertake (i.e., practical information on what needs to be adhered to in the country of delivery)
• Issues: presents related questions and relevant documentation

Apart from national regulations and the corresponding update status, international standards are given as well. Documents are available as .pdf downloads and in English. The user of hSERN can contribute to the resource as well as subscribe to updates of the website and its content.

### International Policy interoperability and data Access Clearinghouse (IPAC)

The ‘International Policy interoperability and data Access Clearinghouse’ (IPAC) is provided by the Public Population Project in Genomics and Society (P3G). Under the IPAC umbrella, P3G offers the Generic Clauses/Agreements Database (http://www.p3g.org/resources/ipac), which is a free online open access database of generic clauses and template agreements to assist researchers in developing policy and contractual documents to facilitate data sharing both prospectively and retrospectively. It provides a unique searchable database containing organized examples of generic clauses to include in consent or policy documents. It also provides consent form templates that can be customized depending on the research domain, as well as access policies, data access agreements, sample transfer agreements, and agreements relating to intellectual property. The database offers approximately 180 generic clauses for six different types of ELSI (Ethical, Legal and Social Implications)-related documents. Additional services provided under IPAC are Ethical and Legal Interoperability Screening and a Data Access Compliance Office (DACO), which provide real-life experts to give advice on specific research projects. For more details, see http://p3g.org/ipac.

An overview of the four tools, which all cover EU law, is provided in Table 1. The tools are characterized by the *material covered* (biosamples or data), the *guidance specificity* (how and on which basis the user is guided), the *tool user* the tool is aiming at, whether *templates* are provided, whether the relevant *legislative framework* is *referenced,* and whether the tool offers *automated guidance* (actively guiding the tool user through questions).

**Table 1.** Key Features of Tools (BioMedBridges LAT, BBMRI legal WIKI, IPAC, hSERN)

	*Features*	*BMB LAT*	*BBMRI wiki*	*IPAC*	*hSERN*
Material covered	Biosamples	•	•		•
	Data	•		•	
Guidance specificity	Guidance based on specific characteristics of sample/data	•		•	
	In-person expert advice available (follow-up)			•	
	Specific requirements or risks for the research context	•		•	
Tool user	Data/sample user		•	•	
	Data/sample provider	•	•	•	
	Legal and/or ethics advisers, support staff	•	•	•	•
Templates provided (consent, participant information)		•	•	•	
References relevant legislative framework		•	•	•	•
Automated guidance		•			

One of the most obvious differences between the tools is the different levels of previous knowledge and expertise required by the user. In addition, the tools are intended for use at different stages of the data or sample sharing process. For example, users may want to check legal and ethical feasibility while applying for project funding, during the implementation of technical infrastructure or the development of data access policies, or when considering how they may share sensitive data. None of the tools covers the use of data and samples in all possible circumstances; rather, they inform the user about general legal/regulatory/ethical considerations. To address specific questions on individual cases, the P3G-IPAC supports users by providing contact information for an expert trained in data protection law whom the user can contact for detailed follow-up discussions. In contrast, the BioMedBridges tool simply highlights potential problem areas that will need follow-up with a legal expert. While most tools leave the user to browse and discover relevant information by themselves, the BioMedBridges tool guides the user through an automated workflow that does not assume any prior knowledge or even awareness of the possible ethical and legal requirements surrounding their use of human research materials.

### Templates

Two of the tools, the P3G-IPAC and the BioMedBridges, provide templates for consent, research participant information, and material or data transfer agreements. While material and data transfer agreements are relatively common in the research environment, providing useful research participant information sheet and consent form templates is more difficult for a variety of reasons. First, it is impossible to foresee the scope of all possible future research purposes; consequently, no single suggestion can be made on what the information sheet and consent form may need to cover. Second, these documents will be presented to the responsible Research Ethics Committee (REC) in order to get approval and, even within the Member States of the European Union, the requirements set by local RECs may differ considerably. This fragmentation of requirements leads not only to a duplication of effort and costs, but also to an increase in administrative burden and, in some cases, may lead to delays or even cancellation of useful projects.

Template providers have taken on the challenges highlighted above and provide templates that are generic to the extent that varying research purposes and legal uncertainties are accounted for. The templates help in ensuring that the current intended use of the samples/data is covered by the scope of the consent under present applicable law. Having appropriate consent from research participants is crucial to any research project except where legislative exemptions exist. As the concept of broad consent for medical research is gaining ground, this type of template may assist in making future use of biosample(s)/data possible.

Publishing templates that are acceptable to all partners of a given research project in different European countries assists in providing additional support in securing a researchers' legal relationship with the research participant.

## Discussion

It is reasonable to suppose that biomedical researchers cannot be expected to have detailed knowledge about all ethical and legal requirements concerning the use of sensitive data and samples in research. Even researchers who have had basic training or previous experience cannot always have in-depth, up to date knowledge of complex and changing requirements which are under constant discussion even among legal experts.

Consequently, tools that aim at assisting the sharing of sensitive data and samples can make researchers aware of their responsibilities and legal requirements in a way that is intuitive and actionable for them. Without prior detailed knowledge, researchers cannot anticipate questions that may need to be asked or requirements that may need to be met. Providing information in a structured, guided way can address this knowledge gap; however, as no individual tool can possibly address all possible scenarios, researchers should be pointed in the direction of experts who can help them understand the issues which arise, particularly in complex situations.

If we were to make a distinction between clinical and basic research in the biomedical context, we would find that in clinical studies, general legal and ethical issues may be addressed in guidelines such as the ICH's ‘Good Clinical Practice’^41^ and the EU Clinical Trials Directive,^42^ therefore, clinical researchers are generally aware of the regulatory implications of the research they are conducting. In the clinical context, a legal assessment tool should focus on concrete solutions as well as provide templates. In basic research scenarios, there is frequently no standard way of handling sensitive data that is intended to be shared in multi-center projects.

Here, it is important to provide high-level guidance to researchers that enables informed decisions to be made, such as when there is a need to consult a legal expert or whether it might be more suitable to anonymize data before making it available, rather than providing pseudonymous data (see *The European regulatory context for data and sample sharing* section for a discussion of relevant considerations linked to anonymization). Of course, a tool might also inform users about suitable algorithms and technical tools that facilitate data anonymization. In general, the suitability and scope of any tool must be assessed and monitored using specific use cases.

A web-based automated tool seems to be the most useful option especially for researchers working in a basic biomedical research setting. However, such an automated tool has inherent limitations: first, while the query process must be based on a set of assumptions with respect to possible use cases, the questions cannot be too specific if the tool is aimed to be generally useful. Second, the content of such a tool can only reflect the high-level, consolidated legal status at any given time. Follow-up expert advice on specific issues, including, for example, the usual practices of local ethics committees or data protection authorities, will almost always be necessary. For any tool, it is important to bear in mind that technical privacy and confidentiality questions, for example, related to the possibility that genomic data might be extrapolated or personally identifiable data matched with metadata from social media, cannot be answered by legal experts.

Even though a tool may show the researcher the “green light,” this will only apply to very straightforward situations. In the majority of cases, the tool will highlight potential issues but such an outcome is still inherently useful: in our view, it would be a huge step forward for many international projects to be able to identify risks with regard to data protection issues early on prior to sharing data or samples. Identifying relevant legal issues and corresponding risks helps in the planning phase of a research study; for example, to know the type of consent from research participants/donors which is needed can be crucial in the development of the study protocol and in planning future work. In some cases, once made aware of possible issues, researchers might be able to adjust their project plans in order to work with anonymized data (but see Section 2 for a discussion of the issues). Highlighting risks before the start of a study means that legal departments can be involved at an early stage in order to find a way of avoiding or resolving substantial issues and risks at the planning stage.

## Outlook

The discussion in our workshop showed that mature tools to support sharing of sensitive samples and data are still lacking. The main challenges to developing tools were identified as:

*ELSI interoperability between countries:* Each country has different legal and ethical contexts; therefore, solutions from one country cannot be mapped 1:1 to another country. A mature tool has to account for this fragmentation by pointing to relevant differences and suggesting practical solutions.

*Definition of relevant use cases:* Any tool has to be developed based on specific assumptions and use cases. In order to maximize the benefit of the tool for the research community, it is essential that context-dependent use cases are defined.

*Dissemination of relevant information:* It is not sufficient to simply provide information and resources and wait for users to discover them. Instead, concentrated efforts are needed to disseminate information about tools and resources to the research community.

It is unlikely or even impossible that one tool will be able to sufficiently cover all use cases. Instead, tools aimed at supporting researchers with ethical and legal issues should focus on specific use cases in order to be of relevance for researchers in that context. Consequently, to capitalize on such discipline-specific expertise and to maximize support to the wider research community, tools should be interoperable and, where appropriate, embed solutions and information from other resources. For example, the detailed legal information in the BBMRI-WIKI might be referenced from the BioMedBridges tool.

Further, it is important to have *quality metrics* that can be used to assess the usefulness of the tool. Access statistics are only an indirect quality measure. While surveys might contribute to determining and refining user/target groups, extremely useful insights that might contribute to the improvement of the tool and its content could be gained by tracking successful applications for data or sample access that directly benefited from the use of the tools, including the use of templates or legal agreements.

The quality of a tool depends on two aspects: content, which requires feedback and input from legal and ethics experts, and technical provision. For both it would be useful to formulate consistent *best practice guidelines,* including comprehensive annotation (e.g., update status, completeness of information, disclaimers—what is covered and what is not, where to get additional information or access expertise) or provision of information in machine-readable format.

Finally, as retired projects might indicate, it is crucial to have a *sustainability strategy* if tools and resources are to survive the project during which they were developed. Even if a tool is provided as an open source solution, responsibility for maintenance at least during the initial period after the end of the project should be clarified. On the technical side, there is no guarantee that the open source community will take up maintenance of a tool and, on the legal side, it is less than likely that experts may freely donate their time to update content. Ideally, funding must be available in order to cover costs for further technical development/adaptation of a tool and dissemination to the user community. It would make sense for these types of tools to be maintained for example by one of the big European research infrastructures that serve relevant scientific communities, such as the new and emerging biomedical science research infrastructures on the ESFRI roadmap.^43^

Equally as important as stable funding is the curation of the tool content. As the legal landscape evolves, legal experts have to be constantly involved in updating the underlying information and assumptions. A general update mechanism must be agreed to and made available, especially in view of the developing situation concerning the EU General Data Protection Regulation. In general, any tool should be designed in such a fashion that updates and extensions are easily made.
